# Poor Sleep Quality and Associated Factors among People Living with HIV/AIDS Attending ART Clinic at Tirunesh Beijing Hospital, Addis Ababa, Ethiopia

**DOI:** 10.1155/2023/6381885

**Published:** 2023-06-16

**Authors:** Atsede Tadesse, Kufa Badasso, Afework Edmealem

**Affiliations:** ^1^Rift Valley University, Addis Ababa, Ethiopia; ^2^Department of Psychiatry, Menelik II Health Science College, Addis Ababa, Ethiopia; ^3^Department of Nursing, Debre Markos University, Debre Markos, Ethiopia

## Abstract

**Background:**

Sleep is a universal need of all higher life forms, including humans. However, sleep problems are one of the most common problems raised by patients living with human immunodeficiency virus/acquired immunodeficiency syndrome (HIV/AIDS). Poor sleep quality is one of the hidden and unrecognized factors contributing to poor medication adherence and functional inactivity among people living with human immunodeficiency virus/acquired immunodeficiency syndrome.

**Methods:**

A hospital-based cross-sectional study was conducted from April 15, 2022, to May 30, 2022, at an antiretroviral therapy (ART) clinic of Tirunesh Beijing Hospital. A systematic sampling technique was used to select study participants. A total of 413 people who are living with human immunodeficiency virus/acquired immunodeficiency syndrome were enrolled in the study. Data were collected through interviews when study participants finished their visit. Variables whose *P* value was less than 0.2 in bivariable logistic regression were entered into multivariable binary logistic regression to identify factors associated with poor sleep quality.

**Result:**

The level of poor sleep quality among people living with HIV/AIDS was 73.7%. People living with HIV/AIDS who had poor sleep hygiene were 2.5 times more likely to have poor sleep quality compared with those patients who had good sleep hygiene. Moreover, study participants who had anxiety were three times more likely to have poor sleep quality compared with those who did not have anxiety (AOR: 3.09; 95% CI = 1.61–5.89). Study participants who had chronic diseases in addition to HIV/AIDS were 3 times more likely to have poor sleep quality compared with those who do not have it (AOR: 2.99; 95% CI = 1.15–7.79). Additionally, people living with HIV/AIDS who were stigmatized due to their disease were 2.5 times more likely to have poor sleep quality compared with their counterparts (AOR = 2.49; 95% CI = 1.43–4.21).

**Conclusion:**

In this study, the magnitude of poor sleep quality among people living with HIV/AIDS was high. Being a farmer, being a merchant, having chronic diseases, having anxiety, having a CD4 count of 200–499 cells/mm^3^, being stigmatized, and having poor sleep hygiene were factors that had an association with poor sleep quality. Healthcare providers should screen people living with HIV/AIDS for anxiety and encourage them to practice good sleep hygiene during follow-up.

## 1. Introduction

Sleep is a universal need of all higher life forms, including humans [1]. Sleep is generated based on a circadian rhythm and homeostatic pressure that follow a period of wakefulness. Sleep helps to modify body temperature, cardiac work, and hormone production, which results in an essential restorative state and proper functioning of the organism [2]. The American Sleep Association recommends that sleep duration for adults should be 7-8 hours [3]. Deprivations of sleep have negative effects on the regulation of weight, sugar, and blood pressure because of the activation of sympathetic nervous system stimulation, systemic inflammation, endothelial dysfunction, and regulation [4, 5]. Despite this fact, the quality of sleep is affected by urbanization, long work schedules, night and shift work, technological advancement, spending more time watching television and using the internet, and disease conditions such as HIV/AIDS [1]. Hence, early recognition of sleep problems via routine assessment and implementation of effective medical and behavioral treatments is necessary to improve functioning and reduce complications [6].

Sleep problems are one of the most common problems raised by patients living with HIV/AIDS. This is because of social stigma and adverse effects of antiretroviral medications, such as increased metabolic disturbance and depression [7], opportunistic infections, and a decrement in the immune system [8]. Studies indicated that the magnitude of poor sleep quality is 40% to 100% among people who are living with HIV/AIDS [9, 10]. Adults who get less than seven or eight hours of sleep can experience mental and physical health deficits [3]. The mental status of PLWHA is often problematic due to depression, anxiety, or other emotional disorders, which are triggered by serious physical symptoms, side effects of medications, financial burdens, stigma, and discrimination [11].

Poor quality sleep can have a negative impact on psychological health and result in an impaired immune system, poor medication adherence, physical inactivity, impaired cell growth and repair, and deteriorated neuronal connections [12, 13]. These increase the health expenses of one's country. In contrast, good sleep quality is associated with better HIV medication adherence, lower HIV symptom severity, and better memory functioning [13]. Poor sleep quality has not only been associated with various diseases but has also led to occupational accidents, poor performance, higher health care utilization, car crash injuries [1], falls, especially in older adults [12, 14], and suicidal ideation [15]. Thus, identifying and treating coexisting sleep problems increases medication adherence, improves the immune system, and slows disease progression [16].

There are different factors, such as the demographic status of the patients, disease characteristics, and substance and alcohol use, that affect the quality of sleep for people living with HIV/AIDS. Among these, because of their bidirectional association, anxiety, and depression are the most common factors associated with poor sleep quality. Poor medication adherence, low CD4 count, type of ART regimen, duration of the disease since diagnosis, and social support are the other prevalent factors associated with poor sleep quality among people living with HIV/AIDS [17–20]. Efavirenz-based regimens are associated with poor sleep quality since efavirenz causes sleep latency and decreases sleep duration [6].

The quality of sleep and its associated factors among people living with HIV/AIDS in Ethiopia are not well studied. To increase medication adherence and quality of life, factors that hinder medication adherence and the immune system, such as poor sleep quality, should be detected and managed. Thus, this study was aimed at assessing the quality of sleep and its associated factors among people living with HIV/AIDS at Tirunesh Beijing Hospital's ART Clinic.

## 2. Methods and Materials

### 2.1. Study Area and Period

The study was conducted at Tirunesh Beijing Hospital. Tirunesh Beijing Hospital, one of Addis Ababa city's public hospitals, was established in 2012. It has served more than 47,982, 4,990, and 8,589 patients in 2020 in outpatient, inpatient, and emergency departments. It has a total of nearly 350 healthcare providers and 50 administrative staff. Among the total number of healthcare providers, 216 are nurses, and 107 are physicians. Tirunesh Beijing Hospital has an ART clinic in addition to other departments. There are 1,057 patients who are living with HIV/AIDS and are on follow-up at the ART Clinic of Tirunesh Beijing Hospital. There are 3 nurses and 1 physician working at the ART Clinic. The ART clinic has a total of 3 rooms. The study was conducted from April 15, 2022, to May 30, 2022.

### 2.2. Study Design

A hospital-based cross-sectional study design was conducted.

### 2.3. Population

All people who are living with HIV/AIDS and are on follow-up at the ART clinic of Tirunesh Beijing Hospital were the source population. All people who were living with HIV/AIDS and on follow-up at the ART clinic of Tirunesh Beijing Hospital during the data collection were considered the study population.

### 2.4. Eligibility Criteria

All people who are living with HIV/AIDS and whose age is 18 years or older were included. On the other hand, the seriously ill and those who are unable to communicate were excluded from the study.

### 2.5. Sample Size Determination

Sample size calculation.

The sample size was calculated using the single population proportion formula with a 95% confidence level, 5% margin of error, and proportion of poor sleep quality among patients living with HIV/AIDS. Proportion, which is 57.6%, was taken from the study conducted on Hawassa [21].

(1) $$\begin{array}{c} \frac{N={\left(Z_{a/2}\right)}^{2}\left(p\right)\;\left(1-p\right)}{d^{2}}\text{,} \end{array}$$

where *N* : sample size, *Z*_*a*/2_ = 1.96 (standardized normal distribution curve value for the 95% confidence interval), *P*=0.576 (proportion of poor sleep quality), and *D* = 0.05 (degree of margin of error) = (1.96)2 (0.576) (0.424)(0.05) 2 = 375.

Since the largest sample was obtained by the first objective, by adding 10% nonresponse rate of calculated sample size, the final sample was 413.

### 2.6. Sampling Procedure

The study utilized the systematic random sampling technique. Study participants were selected in every K value. K value was calculated by dividing the total number of people living with HIV/AIDS at the Tirunesh Beijing Hospital ART clinic to the total sample size. The first study participant was selected using the lottery method from the first comers. K value was calculated as follows:

(2) $$\begin{array}{c} K=\frac{\left(Total\;number\;of\;People\;living\;with\;HIV\right)/AIDS}{Total\;sample\;size}=\frac{1013}{413}=2.45∼2\text{.} \end{array}$$

## 3. Study Variables

### 3.1. Dependent Variables

Sleep quality (good vs. poor).

### 3.2. Independent Variables

- Sociodemographic and economic characteristics: sex, age, marital status, educational status, occupation, address, monthly family income, and body mass index
- Disease characteristics: duration of disease, medication adherence, medication type, recent CD4 count, WHO stage, and the presence of chronic disease,
- Psychosocial: anxiety, depression, stigma, and social support
- Substance use: current use of alcohol, cigarette smoking, chewing tobacco, shisha, or caffeinated drinks
- Physical activity
- Sleep hygiene.

## 4. Data Collection Tool and Procedure

### 4.1. Data Collection Tool

Data were collected using a structured questionnaire, and a face-to-face interview was used for data collection. The tool had three parts. The first part focused on sociodemographic status; the second part assessed the quality of sleep; and the third part assessed factors associated with poor sleep quality, such as anxiety, depression, physical activity, and sleep hygiene. Sleep quality was measured by the Pittsburgh Sleep Quality Index, which is a standardized tool [22]. It has 19 items with seven components. Component 1 is subjective sleep quality; component 2 is sleep latency; component 3 is sleep duration; component 4 is habitual sleep efficiency; component 5 is sleep disturbances; component 6 is the use of sleep medicine; and component 7 is daytime dysfunction. Factors such as anxiety and depression were assessed by the Hospital Anxiety and Depression Scale (HADS). HADS has an anxiety subscale and a depression subscale. Sleep hygiene was assessed by the Sleep Hygiene Index (SHI). It is a tool that has 13-item self-report measures designed to assess the practice of sleep hygiene behaviors. Each item is rated on a five-point scale ranging from 0 (never) to 4 (always), with a total score of 0 indicating good sleep hygiene and 52 representing poor sleep hygiene. The Oslo Social Support Scale (OSS) was used to assess the respondent's support system which has 3 items. The HIV/AIDS-related stigma scale was used to assess stigma. It is a 12-item screening tool. All parts of the questionnaire were initially prepared in English version and translated into Amharic, then back to English to check their consistency.

### 4.2. Data Collection Procedure

After preparing the questionnaire, 3 nurses who have bachelor's degrees for data collection and a master's degree for supervisory duties were recruited. Two days of training were given for each of them on the meaning of every item of the questionnaire and the techniques of data collection, such as ways of greeting, ways of taking consent, ways of monitoring data quality, and ways of addressing ambiguous items. After this, data were collected through face-to-face exit interviews. To prevent repeated interview, the data collectors verified whether the client had been interviewed before or not. Additionally, a chart review was conducted to collect data about WHO stage, CD4 count, type of ART medication, height, and weight. The supervisor and principal investigator closely monitored the data collection process.

### 4.3. Operational Definition

#### 4.3.1. Poor Sleep Quality

The global score of the PSQI is calculated based on the PSQI scoring manual. Then, the global score was categorized into poor sleep quality and good sleep quality. Participants who scored above 5 on the Pittsburgh Sleep Quality Index were categorized as having poor sleep quality [23].

Poor sleep hygiene: the median score was computed. Participants who score above or equal to the median score of the sleep hygiene index [24]. Anxiety: individuals with a sum score of ≥8 from the total sum score will be considered as screened positive for anxiety [24, 25].

Depression: individuals with a sum score of ≥8 from the total sum score will be considered as screened positive for depression [24, 25].

#### 4.3.2. Stigmatized

After computing the mean score, participants who scored above the mean score of HIV/AIDS-related stigma were categorized as stigmatized [26].

#### 4.3.3. Social Support

This category is classified as poor social support, intermediate, and strong if participants scored 3–8, 9–11, or 12–14, respectively [21].

### 4.4. Data Quality Control

The quality of the data was assured at different steps. The first measure taken to assure data quality is the adoption of a standardized questionnaire. After adoption, it was translated into Amharic and back to English for consistency by experts. This is helpful to access quality and accurate data from study participants. Additionally, the data were collected by trained data collectors, and strict supervision was performed while the data were collected. At the end of each data collection, the questionnaires were checked by supervisors for completeness. The second measure was during coding time, during data entry into software, and during analysis. The quality of the collected data was reviewed and checked during the data cleaning process by running simple frequency tests after data entry for its consistency. Inconsistent records were checked by referring to the hard copy questionnaire. Moreover, a pretest was made on 5% of the total sample size at Zewuditu Memorial Hospital two weeks before the actual data collection period.

### 4.5. Data Processing and Analysis

After data collection, completely collected, recoded, and cleaned data were entered into Epidata version 4.1 and exported to Statistical Package and Service Product (SPSS) version 26 for analysis. During analysis, frequency, percentage, and other descriptive statistics were used. The results of the study were presented using text, tables, and figures. The chi-square test and binary logistic regression model were enrolled by considering the 95% confidence level and a *P* value of 0.05. Multivariable binary logistic regression was done by taking variables that have a *P* value of ≤0.2 from bivariable logistic regression to identify factors associated with poor sleep quality. Multivariable logistic regression was enrolled by using the backward likelihood ratio method. The Hosmer and Lemeshow test was utilized to check whether the data fit the model or not before data analysis. The presence of multicollinearity and the assumption of the chi-square test were checked.

### 4.6. Ethical Consideration

Ethical clearance was obtained from the Addis Ababa Public Health Research and Emergency Management Directorate prior to starting data collection (Reference No.: A/A/12855/227; Date: 29/07/2014.C). Then, a supportive letter was given to Tirunesh Beijing Hospital. The purpose and importance of the study were explained, and informed consent was obtained from each participant. Confidentiality was maintained at all levels of the study. To maintain confidentiality, the names of respondents were not registered. Participants' involvement in the study was on a voluntary basis; participants who are unwilling to participate in the study and those who wish to quit their participation at any stage will be informed to do so without any restriction.

## 5. Result

### 5.1. Sociodemographic Characteristics of Respondents

A total of 413 study participants were enrolled in this study. Among these, 388 completed the questionnaire, with a response rate of 93.94%. From a total of 388 study participants, 211 (54.4%) were female. The mean age of respondents was 39.78 (SD–9.7), and 351 (90.5%) of them were from 25 to 55 years of age. Among the total study participants, 117 (30.2%) were single in their marital status; 103 (26.5%) of them had no permanent job; 79 (20.4%) lived in rural areas; and 58 (14.9%) were farmers. A total of 162 (41.8%) study participants had an educational status of college or above. Slightly above one-tenth (11.6%) of the total study participants had a body mass score of less than 18.5 kg/m^2^ (Table 1).

### 5.2. Disease-Related Characteristics

Of the total study participants, the duration of their disease was above five years for 190 (49%), and the stage of their disease was above stage one for 72.2% of them. Over one-fifth of the total study participants took second line antiretro viral drugs (ART), and only 5 (1.3%) of them took an efavirenz-based regimen. The recent CD4 count for 78.6% of study participants was less than 500 cells/mm^3^. Additionally, of the total study participants, nearly one-third (32.7%) had poor or fair drug adherence (Table 2).

### 5.3. Substance Use

Among the total study participants, 67 (17.5%) of them had smoked cigarettes in the past month, and 153 (39.4%) of them drank alcohol. Additionally, one-fifth (25.5%) of the total study patients had a history of chewing tobacco for the past month (Table 3).

### 5.4. Psychosocial Factors, Sleep Hygiene, and Physical Activity

Among the total study participants, 329 (84.8%) had anxiety and 351 (90.5%) had depression. Two hundred and forty respondents were stigmatized, and 239 (61.6%) got poor social support. A total of 199 (51.3%) respondents had poor sleep hygiene. From a total of 155 study participants, 33 (21.9%) of them did not have health-enhancing physical activity (Table 4).s

### 5.5. Quality of Sleep by the Pittsburgh Sleep Quality Index (PSQI) Subscale

From the total of 388 study participants, only 30 (7.7%) reported their sleep quality as very good, 250 (64.4%) of them fell asleep within 30 minutes, and more than three-fourth of them slept the recommended sleep duration. Moreover, 352 (90.7%) respondent slept for at least 85% of the time they were in bed. The quality of sleep for 248 (64%) of the study participants was disturbed at least once a week and above. Sleep disturbances kept 324 respondents from performing their daytime tasks (Table 5).

### 5.6. Overall Sleep Quality

Among the total 388 respondents, 286 (73.7%) of them had reported poor sleep quality.

### 5.7. Factors Associated with Sleep Quality

Variables that had an association with sleep quality at a *P* value ≤ 0.2 in bivariable logistic regression were sex, occupation, body mass index, presence of chronic disease, smoking, alcohol, chewing tobacco, taking substances such as shisha, recent CD4 count, anxiety, depression, sleep hygiene, social support, and stigma. However, in multivariable logistic regression, only occupation, presence of chronic disease, recent CD4 count, anxiety, sleep hygiene, and HIV-related stigma were associated with sleep quality at a *P* value of 0.05. Accordingly, respondents whose occupation was farming were 5 times more likely to develop poor sleep quality compared with students (AOR: 4.79; 95% CI = 1.30–17.56). On the other hand, study participants who had chronic diseases in addition to HIV/AIDS were 3 times more likely to have poor sleep quality compared with their counterparts (AOR: 2.99; 95% CI = 1.15–7.79). Additionally, people living with HIV/AIDS who had anxiety were 3 times more likely to have poor sleep quality compared with those who did not have anxiety (AOR: 3.09; 95% CI = 1.61–5.89). Similarly, people living with HIV/AIDS who were stigmatized due to their disease were 2.5 times more likely to have poor sleep quality compared with their counterparts (AOR = 2.49; 95% CI = 1.43–4.21) (Table 6).

## 6. Discussion

The quality of sleep, especially in patients with chronic illness, should be assessed since it affects cognitive, physical, and psychosocial health in a multidimensional way [27]. Patients with HIV/AIDS were affected by poor sleep quality for different reasons. Hence, this study was done to assess the quality of sleep among HIV/AIDS patients and its associated factors in the Tirunesh Beijing Hospital ART clinic.

In this study, the quality of sleep among patients with HIV/AIDS was 73.7% (95% CI: 69.3%–78.1%). This finding is higher than the finding of previous studies conducted in Hawassa, South Ethiopia (57.6%) [21], Metu Ethiopia (57.1%) [23], and Zewuditu Memorial Hospital (55.6%) [26]. Similarly, the findings of this study are higher than those of studies conducted abroad [6, 17, 18].

According to the findings of the study, people living with HIV/AIDS whose occupation was farming were five times more likely to have poor sleep quality compared with those who were students. This might happen because of poor sleep hygiene and uncomfortable living conditions. Although there are limited findings that support these findings, an association between poor sleep quality and employment status was observed in the previous study [28].

People living with HIV/AIDS who had a CD4 count of 200–499 cells/mm^3^ were 2.69 times more likely to have poor sleep quality compared with those whose CD4 count was less than 200 cells/mm^3^. The possible justification for this might be due to the presence of different opportunistic infections and chronic illnesses. According to the findings of this study, the prevalence of chronic illnesses was high among those study participants whose CD4 count was 200–499 cells/mm^3^. This finding is supported by a study conducted in South Africa [29].

HIV-positive patients are vulnerable to depression and anxiety as psychological disorders [28]. In this study, people living with HIV/AIDS who had anxiety were three times more likely to have poor sleep quality compared with their counterparts. This might be due to the bidirectional association between poor sleep quality and anxiety [30]. The sleep-wake cycle is regulated mainly by the hypothalamus. When there is anxiety, the function of the hypothalamus pituitary axis is disturbed [31]. On the contrary, poor sleep quality results in excessive discharge of the sympathetic nervous system, which in turn results in anxiety and stress. This finding is supported by a study conducted at Zewuditu Memorial Hospital [26], Hawassa [21], and China [18].

People living with HIV/AIDS and who have had chronic illnesses were three times more likely to have poor sleep quality compared with those who did not. The possible justification for this might be the double burden of chronic illnesses and HIV/AIDS. This finding is supported by a study conducted in Hawassa [21], Zewuditu Hospital, Addis Ababa [26].

Furthermore, people living with HIV/AIDS who were stigmatized due to their illness were 2.5 times more likely to have poor sleep quality compared with their counterparts. This might be due to the fact that people living with HIV/AIDS who experience HIV-related stigma may experience greater feelings of loneliness, which are related to increased depressive symptoms [32]. This finding is supported by a study conducted at Zewuditu Memorial Hospital [26].

More interestingly, people living with HIV/AIDS who had poor sleep hygiene were 2.57 times more likely to have poor sleep quality compared with those who had good sleep hygiene. The possible justification for this might be that good sleep habits and practices promote quality sleep. This finding is supported by a study conducted at Zewuditu Memorial Hospital [26] and in India among cancer patients [32].

## 7. Conclusion

In this study, the magnitude of poor sleep quality among people living with HIV/AIDS was high. Being a farmer, being a merchant, having additional chronic diseases, having anxiety, having CD4 count of 200–499 cell/mm^3^, being stigmatized, and having poor sleep hygiene were factors that had an association with poor sleep quality among people living with HIV/AIDS. Healthcare providers work hard, counsel, and advise to prevent the emergence of chronic illnesses. Additionally, during follow-up, people living with HIV/AIDS should be screened for anxiety regularly, and appropriate treatment should be provided. People living with HIV/AIDS who have low CD4 and whose occupation is farmer or merchant should get special support. The Ministry of Health should expand healthcare providers who promote good sleep hygiene, particularly for people living with HIV/AIDS. Additionally, the Ministry of Health should minimize HIV-related stigma among the public through different strategies.

## Figures and Tables

**Table 1:** Sociodemographic status of patients with HIV/AIDS at Tirunesh Beijing Hospital ART Clinic, 2022 (*N*-388).

Variables	Category	Frequency	Percentage
Sex	Female	211	54.4
	Male	177	45.6

Age	18–24	8	2.1
	25–55	351	90.5
	56–64	22	5.7
	≥65	7	1.8

Educational level	Unable to read and write	22	5.7
	Able to read and write (informal school)	113	29.1
	Grade 1–8	28	7.2
	Grade 9–12	63	16.2
	College and above	162	41.8

Marital status	Single	117	30.2
	Married	124	32
	Widow	81	20.9
	Divorced	66	17

Residence	Urban	309	79.6
	Rural	79	20.4

Occupation	Farmer	58	14.9
	Merchant	99	25.5
	Student	21	5.4
	Employee	107	27.6
	Others (retired, no permanent job)	103	26.5

Monthly income (ETB)	≤2225	97	25
	2226–3650	97	25
	3651–5500	101	26
	>5500	93	24

BMI (kg/m^2^)	<18.5	45	11.6
	18.5–24.99	249	64.2
	>25	94	24.2

Monthly income was categorized based on quartile range; BMI was based on WHO weight classification for Ethiopia.

**Table 2:** Disease characteristics among patients with HIV/AIDS at Tirunesh Beijing Hospital ART Clinic, 2022 (*N*-388).

Variables	Category	Frequency	Percentage
Duration of disease after diagnosis	5 years and below	198	51
	Above 5 years	190	49

WHO staging	Stage I	108	27.8
	Stage II	195	50.3
	Stage III	84	21.6
	Stage IV	1	0.3

ART type	Non-Efavierenz	299	77.1
	Efavierenz-based	5	1.3
	2^nd^ line treatment	84	21.6

CD4 count	<200	52	13.4
	200–499	253	65.2
	≥500	83	21.4

Presence of chronic disease	Yes	62	16
	No	326	84

Adherence to medication	Good	261	67.3
	Fair	78	20.1
	Poor	49	12.6

**Table 3:** Substance, alcohol, and caffeine use among patients with HIV/AIDS at Tirunesh Beijing Hospital ART Clinic, 2022 (*N*-388).

Variables	Category	Frequency	Percentage
Coffee and/or tea	Yes	324	83.5
	No	64	16.5

Cigarette smoking	Yes	67	17.5
	No	321	82.7

Alcohol drinking	Yes	153	39.4
	No	235	60.6

Chat chewing	Yes	99	25.5
	No	289	74.5

Using shisha and other substances	Yes	85	21.9
	No	303	78.1

**Table 4:** Psychosocial factors, sleep hygiene, and physical activity among patients with HIV/AIDS at Tirunesh Beijing Hospital ART Clinic, 2022 (*N*-388).

Variable	Category	Frequency	Percentage
Anxiety	Have no anxiety	59	15.2
	Have anxiety	329	84.8

Depression	Have no depression	37	9.5
	Have depression	351	90.5

HIV related stigma	No	148	38.1
	Yes	240	61.9

Social support	Poor social support	239	61.6
	Intermediate social support	91	23.5
	Strong social support	58	14.9

Sleep hygiene	Good	189	48.7
	Poor	199	51.3

Physical activity	Inactive	0	0
	Minimally active	33	21.9
	HEPA	122	78.1
	Total	155	100

**Table 5:** Quality of sleep by the Pittsburgh sleep quality index (PSQI) subscale among patients with HIV/AIDS at Tirunesh Beijing Hospital ART Clinic, 2022 (*N*-388).

PSQI subscale	Category	Frequency	Percentage
Subjective sleep quality (component 1)	Very good	30	7.7
	Fairly good	119	30.7
	Fairly bad	162	41.8
	Very bad	77	19.8

Subscale of sleep latency (component 2)	0–15 minutes	32	8.2
	16–30 minutes	218	56.2
	31–60 minutes	121	31.2
	>60 minutes	17	4.4

Sleep duration (component 3)	≥7 hours	296	76.3
	6-7 hours	64	16.5
	5-6 hours	23	5.9
	Less than 5 hours	5	1.3

Habitual sleep efficiency (component 4)	≥85%	352	90.7
	75–84%	24	6.2
	65–74%	1	0.3
	Less than 65%	11	2.8

Sleep disturbance (component 5)	Not disturbed in past one month	9	2.3
	Less than once a week	131	33.8
	Once or twice a week	235	60.6
	Three or more times a week	13	3.4

Medication use for sleep (component 6)	Not during the past month	213	54.9
	Less than once a week	83	21.4
	Once or twice a week	58	14.9
	Three or more times a week	34	8.8

Day time dysfunction (component 7)	Not during the past month	74	19.1
	Less than once a week	106	27.3
	Once or twice a week	172	44.3
	Three or more times a week	36	9.3

**Table 6:** Bivariable and multivariable logistic regression outputs on the association between sleep quality and factors, 2022 (*N* = 388).

Variables	Category	*Sleep quality*		COR at 95% CI	AOR at 95% CI
		Poor	Good		
Sex	Female	150	61	0.74 (0.46–1.17)	
	Male	136	41	1	

Occupation	Farmer	47	11	3.20 (1.08–9.48)	4.79 (1.30–17.56)
	Merchant	79	20	2.96 (1.09–8.07)	4.84 (1.46–16.03)
	Student	12	9	1	1
	Employee	76	31	1.83 (0.70–4.80)	2.76 (0.87–8.08)
	Others (retired…)	72	31	1.74 (0.66–4.55)	3.06 (0.95–9.81)

Presence chronic ds	Yes	56	6	3.89 (1.64–9.34)	2.99 (1.15–7.79)
	No	230	96	1	1

Smoking cigarette	Yes	56	11	2.01 (1.01–4.01)	
	No	230	91	1	

Alcohol drinking	Yes	120	33	1.51 (0.93–2.45)	
	No	166	69	1	

Chat chewing	Yes	78	21	1.44 (0.83–2.49)	
	No	208	81	1	

Using shisha and others	Yes	73	12	2.57 (1.33–4.96)	
	No	213	90	1	

Recent CD4 count (cells/mm^3^)	<200	27	25	1	1
	200–499	197	56	3.25 (1.75–6.05)	2.69 (1.34–5.42)
	≥500	62	21	2.73 (1.31–5.07)	2.20 (0.98–5.07)

Anxiety	Had no anxiety	27	32	1	1
	Had anxiety	259	70	4.38 (2.46–7.80)	3.09 (1.61–5.89)

Depression	Had no depression	24	13	1	1
	Had depression	262	89	1.59 (0.77–3.26)	

Sleep hygiene	Good	113	76	1	1
	Poor	173	26	4.47 (2.70–7.41)	2.57 (1.44–4.41)

BMI (kg/m^2^)	<18.5	43	2	8.07 (1.90–34.26)	
	18.5–24.99	181	68	1	
	>25	62	32	0.72 (0.43–1.21)	

Social support	Poor	175	64	1.43 (0.78–2.65)	
	Intermediate	73	18	2.13 (1.01–4.51)	
	Strong	38	20	1	

HIV-related stigma	No	88	60	1	1
	Yes	198	42	3.21 (2.01–5.13)	2.49 (1.43–4.21)

## Data Availability

The dataset will not be shared in order to protect the participants' identities but is available from the corresponding author on reasonable request.

## Abbreviations

AIDS: Acquired immune deficiency syndrome
ART: Antiretroviral therapy
ETB: Ethiopian birr
HIV: Human immune deficiency virus
HADS: Hospital anxiety and depression scale
MDGs: Millennium development goals
OR: Odds ratio
PLWHA: People living with HIV/AIDS
SHI: Sleep hygiene index
SPSS: Statistical package for social science
WHO: World Health Organization
